# Public (un)willingness to trade-off benefits and harms in decisions about cancer screening: A discrete choice experiment

**DOI:** 10.1177/09691413261422933

**Published:** 2026-03-05

**Authors:** Rebecca A Dennison, Stuart Wright, Georgios Lyratzopoulos, Nora Pashayan, Stephen Morris, Brian Nicholson, Thiscovery Authorship Group, Juliet A. Usher-Smith

**Affiliations:** 1 Department of Public Health and Primary Care, 2152 University of Cambridge, Cambridge, UK; 2 Health Services Research and Primary Care, School of Health Sciences, 5292 University of Manchester, Manchester, UK; 3 Research Department of Behavioural Science and Health, 4919 University College London, London, UK; 4 Nuffield Department of Primary Care Health Sciences, 6396 University of Oxford, Oxford, UK

**Keywords:** Discrete choice experiment, early detection of cancer, health policy, mass screening, overdiagnosis

## Abstract

**Objectives:**

To quantify the harms participants are willing to accept for varying benefits from cancer screening following provision of information.

**Methods:**

We conducted an online discrete choice experiment with two samples of the UK public. Attributes were number of cancer diagnoses and deaths prevented (benefits) and overdiagnoses, false positives and false negatives (harms) for a population who are/are not screened for an unspecified cancer over their lifetimes. After selecting the best-fitting conditional logistic regression model for each sample, we used latent class analysis to investigate preference heterogeneity and calculated the probability that each class/group would take up screening with different benefit and harm levels.

**Results:**

In total, 1018 participants completed the discrete choice experiment. Cancer deaths prevented was the most important attribute, followed by the number of false positively/negatively detected cancers; cancers prevented and overdiagnosed were deemed relatively unimportant. Three latent classes were identified. The largest class (approximately two-thirds) chiefly opted for screening, focusing on deaths prevented; this group would strongly prefer screening participation even with harms only (probability 76%–92%). Approximately a quarter of each sample traded off benefits and harms; this group would likely take up screening with outcomes like those of colorectal screening (68%–87%).

**Conclusions:**

A minority of the public express preferences for screening that are influenced by trading-off potential benefits/harms, but most have strong preferences for cancer screening, focusing on the associated reduction of cancer deaths. The findings emphasise public health responsibility in appraising or generating evidence on benefits and harms to support decisions about the introduction or modification of screening programmes.

## Introduction

Screening aims to identify early-stage cancer when it is often easier to treat successfully and, for some cancers, to prevent occurrence by intercepting pre-malignant lesions. Screening for cervical, breast and colorectal cancer has been estimated to prevent around 9000 deaths each year in England,^1^ with targeted lung cancer screening rolled out recently.^2^ Additionally, screening brings other less measurable benefits, including reassurance from a negative result and recognition of an individual's value to society.^3^

However, screening can also result in psychological, physical and financial harms and opportunity costs. Some harms are associated with the screening test itself, such as pain, exposure to radiation or anxiety. Others arise from inaccuracies in the result: false positives in which people undergo further testing when they do not have cancer and, more rarely, false negatives in which a cancer is missed and diagnoses delayed. In addition, overdetection and overtreatment occur where a cancer that would not have caused harm over the lifetime is diagnosed and treated (referred to by the broader term ‘overdiagnosis’ throughout).

Despite growing awareness of the harms of screening, particularly among healthcare professionals and researchers, surveys of public attitudes show high enthusiasm about screening.^4,5^ For example, almost 90% of UK participants sampled in 2012 thought that screening is ‘almost always a good idea’ (*n* = 1682/2024),^4^ and more recently, the number of deaths prevented through screening was more than three times more important to respondents than the number of people harmed by further investigations.^6^

Other research suggests that support remains even when participants are told that screening will not benefit them in terms of cancer outcomes: 49% (*n* = 931/2024) said they would be tested for cancer even if it was untreatable^4^ and another 33% (*n* = 293/877) who believed that a hypothetical test does not save lives wanted it anyway.^7^ Elsewhere, participants were willing for 113–313 people to experience overdetection per 1000 screened.^8^ However, these and other surveys tend not to require consideration of multiple different benefits and harms over time, nor do participants need to trade potential benefits against harms. Given frequent changes in cancer screening, including new screening programmes and tests (such as lung screening and multicancer detection tests), exploring public perspectives to inform polices and communication is increasingly important.

We aimed, therefore, to quantify the harms that people are willing to accept for varying benefits from cancer screening, following the provision of information about lifetime impacts. Secondary aims were to estimate the proportion of people who are likely to take up screening at different levels of benefits and harms and to identify characteristics of groups with similar priorities.

## Methods

To address these aims, we designed a discrete choice experiment (DCE). DCEs enable quantification of preferences for different characteristics (attributes) by asking participants to select between hypothetical options in a series of questions.^9^

### Survey design

#### Survey structure

The survey (Supplementary File 1) consisted of: (a) beliefs about cancer screening, reported separately; (b) background information and the DCE; (c) an evaluation of the DCE process including its ease/difficulty, decision-making strategy and ranking the attributes; and (d) demographics, measures of lifestyle and screening history.

The DCE background information included an explanation of the purpose of the study, an overview of screening in the UK, and details of screening benefits and harms that related to each attribute. Brief explanations of each attribute were also available with the questions.

#### Attributes and levels

We listed important screening benefits and harms based on previous surveys on this topic^6–8,10–14^. Quantifiable attributes and levels (Table 1) were then selected through discussions with experts in cancer screening and DCE design, and patient and public involvement (PPI) representatives. Levels were picked to represent plausible and clinically relevant ranges that could be seen within current breast, colorectal and cervical screening programmes^12,15–26^. They were presented for a population of 1000 people who do and do not undergo a lifetime of screening (e.g. completing a screening test every 2 years for 20 years).

**Table 1.** Description of the attributes and levels used within the DCE, assuming a population of 1000 people never screened or screened regularly over their screening lifetime.

Attribute (simplified label)	Definition	Levels	Example
Cancers diagnosed and prevented (cancers diagnosed)	Screening can *prevent the specific cancer* by finding and treating cell abnormalities before cancer develops. This treatment is likely to be a one-off and may take place during the investigation and not require future appointments.	50 diagnosed - 0 prevented^a^ 49 diagnosed - 1 prevented 48 diagnosed - 2 prevented 44 diagnosed - 6 prevented	49 people will be diagnosed with the cancer; one cancer will be prevented through screening.
Deaths and deaths prevented (cancer deaths)	Screening can *prevent deaths from the specific cancer* through earlier diagnosis and treatment.	10 deaths - 0 prevented^a^ 7 deaths - 3 prevented 5 deaths - 5 prevented 3 deaths - 7 prevented	Five people will die from the cancer; five cancer deaths will be prevented through screening.
Overdetection and overtreatment (overdiagnoses)	Screening can lead to *overdetection and overtreatment*. Some people will be diagnosed with a cancer that would not have made them ill during their lifetime and they have treatment they did not need. They would not know this but would think that screening had saved their life.	0 overdetections^ab^ 2 overdetections 10 overdetections 15 overdetections 30 overdetections	30 people will be diagnosed but the cancer would not have caused them harm or have killed them.
False positives (false positives)	Some people need further investigations, and these show that they do not have cancer. These are known as *false positives*: the screening test was wrong, and they did not actually need to have extra tests that might have been invasive.	0 false positives^ab^ 200 false positives 300 false positives 400 false positives 500 false positives	200 people will have a positive screening test but further investigations show they do not have cancer.
False negatives (false negatives)	Other people receive a negative screening test result when they actually do have cancer. These are known as *false negatives*: they are falsely reassured, which may delay diagnosis and treatment or cause them to dismiss symptoms.	0 false negatives^ab^ 1 false negative 10 false negatives 20 false negatives 50 false negatives	10 people will have a negative screening test when they actually do have cancer.

DCE: discrete choice experiment.

^a^ No screening option: 50 cancers diagnosed - 0 prevented, 10 deaths - 0 prevented, 0 overdiagnoses, 0 false positives, 0 false negatives.

^b^ Used in no screening option only.

#### Experimental design and DCE question

Each DCE question (choice set) comprised two screening options plus no screening (opt-out, with neither associated benefits nor harms). An experimental design maximising the D-efficiency of the design based on the covariance matrix of the conditional logit model and with non-informative (zero) priors was generated using the Stata command -*dcreate-*, and then the questions were split into two sets of nine questions using -*blockdes-*. Participants had to answer each question in a forced choice-elicitation format. The question was: ‘Imagine that you were invited to take part in screening for this cancer. Which option would you choose? Option 1/Option 2/No screening’.

#### DCE visual design

The design of each question was based on best practice for risk communication.^27,28^ Specifically, this included the presentation of the numbers of benefits and harms in both words and a visual icon array with simple labels. We chose a colourblind-suitable palette and avoided using reds or greens that could have implied the attributes’ importance.

#### Piloting and revision

The survey was refined through two 1-h workshops, each with three different PPI representatives. Prior to meeting, they were sent a summary of the project and survey excerpt. Workshops involved general feedback plus discussion of key questions posed by the study lead. The first meeting focused on the background information, and the second focused on DCE question presentation. Important changes included omitting a suggested attribute (deaths attributable to screening, which was deemed of negligible importance due to its low magnitude) and changing the layout of the figure to increase understandability. PPI representatives also recommended that screening harms be omitted from the survey (background information and DCE attributes) completely, which we did not implement. The final design is shown in Supplementary File 1 (Example 1; participants were able to see an explanation of each attribute by clicking on the question mark symbols; the lower visual could be shown/hidden as preferred).

Feedback collected from the first 259 responses (Version 1) showed the background information could be overwhelming, and therefore, we changed the webpage structure to utilise clickable drop-down boxes so participants did not need to see all information at once. Also, we removed the three true/false questions initially included to facilitate and check comprehension as these were considered intimidating and led to drop-out. The remaining participants completed Version 2. Responses from both versions were included in the analysis.

### Participants and recruitment

We aimed to recruit 1000 people over age 18 and resident in the UK who, as far as possible, represented the diversity of the UK adult population in terms of age, sex and ethnicity. We did this via two sampling strategies.

#### Sample A

The initial sample was recruited via Thiscovery (www.thiscovery.org), an online healthcare services engagement, consultation and research platform. The survey was disseminated via the Thiscovery crowd contact email list (≥10,000 registered users), associated social media networks, the personal and professional networks of the research and Thiscovery teams, and other partners. Monetary incentives were not offered to cater to the motivation of the Thiscovery network^29^ and mitigate the risks of fraudulent participation. Sample A completed survey Version 1 or 2.

#### Sample B

We recruited the remaining participants through Prolific (www.prolific.com), an online participant recruitment platform with approximately 70,000 active registered members in the UK, without a healthcare focus. A sample representing the diversity of the UK adult population in terms of age, sex and ethnicity was sought. Sample B received £3 for their participation and completed survey Version 2 only.

#### Consent and data collection

The survey for both samples was hosted on the Thiscovery website. Participant information was available at the start of the survey and was followed by consent. Participants were free to withdraw for any reason at any time.

### Statistical analysis

Participants’ demographics, cancer and screening experiences, perceived risk, and evaluation of the DCE task were summarised using descriptive statistics. Chi-squared tests were used to identify differences between the characteristics of Samples A and B.

The analysis – investigation of heterogeneity, model selection and calculations using the coefficients – is detailed in Supplementary File 2, Table S1, and the code is provided in full (see Data availability statement). Briefly, the logistic regression models that best fit the dataset were first selected for each sample. The relative importance of each attribute (RAI) was calculated using attribute-based normalisation.^30^ Preference heterogeneity and whether the resulting classes can be predicted by participant characteristics was investigated using latent class analysis. Lastly, we calculated the probability that each class would take up screening programmes with different levels of benefits and harms: first, without benefits but different levels of harms; second, with levels comparable to current UK cancer screening programmes (detailed in Table S2). No screening (modelled as the no screening constant, 50 cancers diagnosed, 10 cancer deaths and no harms) was used as the comparator.

All analyses were performed using Stata 15 (StataCorp, College Station, Texas, USA). Microsoft Excel was used to perform calculations using the coefficients.

## Results

### Participant characteristics

The survey was live between September and December 2024 (Sample A: 11 September–16 November; Sample B: 3 December only). In total, 1620 participants opened the survey and 1018 participants completed the DCE; 63 participants declined consent, 465 participants dropped out prior to the DCE, and 74 participants dropped out during the DCE.

Median time to complete the survey was 22:55 min (interquartile range: 15:09–35:34) for Sample A and 15:09 min (10:51–21:09) for Sample B; *p* < 0.001 for difference. Less than 1% (10/1018) completed the survey in less than 5 min. Given this small proportion, all those who completed the survey were included in the analysis.

There were statistically significant differences between the samples for all characteristics (Table 2). Most notably, 22% reported a history of cancer in Sample A versus 5% in Sample B, and 86% had participated in cancer screening compared with 57%, respectively. Sample A also had a higher perceived chance of developing cancer and more frequently worried. Overall, and more so for Sample A, our sample included more females, people of white ethnicity and with higher levels of education and higher perceived socioeconomic status than the UK population.^31^

**Table 2.** Sample characteristics.

	Total sample	Sample A	Sample B
Category (*p*-value^a^)	*N* (%)	*N* (%)	*N* (%)
Total	1018 (100.0)	414 (40.7)	604 (59.3)
*Age range (<0.001)*			
18–24	70 (6.9)	5 (1.2)	65 (10.8)
25–39	200 (19.6)	45 (10.9)	155 (25.7)
40–59	377 (37.0)	142 (34.3)	235 (38.9)
60–79	322 (31.6)	180 (43.5)	142 (23.5)
≥80	29 (2.8)	26 (6.3)	3 (0.5)
Missing (NR)	20 (2.0)	16 (3.9)	4 (0.7)
*Sex (<0.001)*			
Male	377 (37.0)	86 (20.8)	291 (48.2)
Female	628 (61.7)	317 (76.6)	311 (51.5)
Prefer not to say or missing (NR)	13 (1.3)	11 (2.7)	2 (0.3)
*Ethnicity (<0.001)*			
Asian	59 (5.8)	10 (2.4)	49 (8.1)
Black	23 (2.3)	3 (0.7)	20 (3.3)
Mixed	16 (1.6)	6 (1.4)	10 (1.7)
White	895 (87.9)	377 (91.1)	518 (85.8)
Other	7 (0.7)	5 (1.2)	2 (0.3)
Prefer not to say or missing (NR)	18 (1.8)	13 (3.1)	5 (0.8)
*Education (<0.001)*			
Left school at or before the age of 15	14 (1.4)	6 (1.4)	8 (1.3)
Completed GCSEs, O Levels or equivalent	103 (10.1)	29 (7.0)	74 (12.3)
Completed A Levels or equivalent	126 (12.4)	16 (3.9)	110 (18.2)
Further education	127 (12.5)	50 (12.1)	77 (12.7)
Undergraduate degree	385 (37.8)	166 (40.1)	219 (36.3)
Postgraduate degree	243 (23.9)	131 (31.6)	112 (18.5)
Prefer not to say or missing (NR)	20 (2.0)	16 (3.9)	4 (0.7)
*Perceived position on the socioeconomic ladder (<0.001)*			
9–10 (highest)	74 (7.3)	67 (16.2)	7 (1.2)
7–8	388 (38.1)	192 (46.4)	196 (32.5)
4–6	444 (43.6)	117 (28.3)	327 (54.1)
1–3 (lowest)	76 (7.5)	13 (3.1)	63 (10.4)
Prefer not to say or missing (NR)	36 (3.5)	25 (6.0)	11 (1.8)
*Personal history of cancer (<0.001)*			
Yes	119 (11.7)	90 (21.7)	29 (4.8)
No	876 (86.1)	307 (74.2)	569 (94.2)
Prefer not to say or missing (NR)	23 (2.3)	17 (4.1)	6 (1.0)
*Previous cancer screening attendance (<0.001)*			
Yes	700 (68.8)	357 (86.2)	343 (56.8)
No, have not been invited	244 (24.0)	34 (8.2)	210 (34.8)
No, have chosen not to have screening	52 (5.1)	9 (2.2)	43 (7.1)
Prefer not to say or missing (NR)	22 (2.2)	14 (3.4)	8 (1.3)
*Perceived likelihood of developing cancer in the next 10 years (<0.001)*			
Likely (extremely to slightly likely)	600 (58.9)	299 (72.2)	301 (49.8)
Unlikely (neither likely nor unlikely, or slightly to extremely unlikely)	357 (35.1)	90 (21.7)	267 (44.2)
Prefer not to say or missing (NR)	61 (6.0)	25 (6.0)	36 (6.0)
*Frequency of thinking about chance of getting cancer in the past month (<0.001)*			
Not at all, rarely or sometimes	825 (81.0)	295 (71.3)	530 (87.7)
Often or a lot	172 (16.9)	104 (25.1)	68 (11.3)
Prefer not to say or missing (NR)	21 (2.1)	15 (3.6)	6 (1.0)

GCSE: general certificate of secondary education; NR: not reported.

^a^ χ^2^ *p*-value for Sample A versus B.

### Preferences for screening benefits and harms

The model selection process suggested scale heterogeneity between the samples (Table S3.A), and therefore, models for Samples A and B were selected and analysed separately. Additionally, there was evidence of scale heterogeneity between Versions 1 and 2, and therefore, a heteroscedastic conditional logistic regression model was used for Sample A (Tables S3.A and S4.A). The conditional logistic regression model with a piecewise transformation of the cancers diagnosed attribute fitted Sample B best (Table S4.B).

Table 3 shows the principal logistic regression results. Participants in both samples preferred screening over no screening. The more-negative coefficient for no screening in Sample A indicates stronger preferences for screening in Sample A than B. Participants also preferred screening options with the greatest benefits and least harms; namely the fewest people diagnosed with and dying from cancer and the fewest people with overdiagnoses, false positives and false negatives. Each attribute was significantly associated with participant preferences. The piecewise transformation of number of cancer diagnoses in Sample B suggests that this was particularly so when there were >48 per 1000.

**Table 3.** Logistic regression analysis and relative attribute importance.

		Sample A^a^		Sample B^b^	
		Coefficient (95% CI)	RAI (%)	Coefficient (95% CI)	RAI (%)
No screening (ASC)		−1.075 (−1.333 to −0.818)**	NA	−0.445 (−0.624 to −0.267)**	NA
Cancers diagnosed	≤48	−0.070 (−0.094 to −0.046)**	8.3	−0.025 (−0.052 to 0.003)	2.3
	>48			−0.214 (−0.271 to −0.156)**	4.9
Cancer deaths		−0.294 (−0.342 to −0.247)**	40.7	−0.270 (−0.287 to −0.254)**	43.7
Overdiagnoses		−0.012 (−0.018 to −0.006)**	7.0	−0.006 (−0.010 to −0.002)*	4.2
False positives		−0.002 (−0.003 to −0.002)**	22.6	−0.002 (−0.002 to −0.001)**	20.1
False negatives		−0.022 (−0.026 to −0.017)**	21.4	−0.022 (−0.024 to −0.019)**	24.9

ASC: alternative specific constant; CI: confidence interval; NA: not applicable; RAI: percentage relative attribute importance.

Note: Attribute levels presented as n in a population of 1000 people screened regularly over their screening lifetime.

*p < 0.05; **p < 0.001.

^a^ Heteroscedastic logistic regression; 414 participants and 11,178 observations.

^b^ Conditional logistic regression with piecewise transformation of number of cancers diagnosed; 604 participants and 16,308 observations.

Over the range of included levels, cancer deaths prevented was the most important attribute for participants’ choice between options (40.7%–43.7% RAI), followed by number of people with false positives and false negatives (20.1%–24.9% RAI) (Table 3). Conversely, participants considered prevention of cancer and overdiagnosis to be the least important.

### Preference heterogeneity

For both samples, three classes were used as a balance between model fit based on Bayesian information criteria, proportion of participants in each class, and distinction between the views of participants in each class (see Tables S5.A and S5.B). These are referred to as Class 1/2/3 Sample A/B, for example, Class 1A.

**Figure 1.**
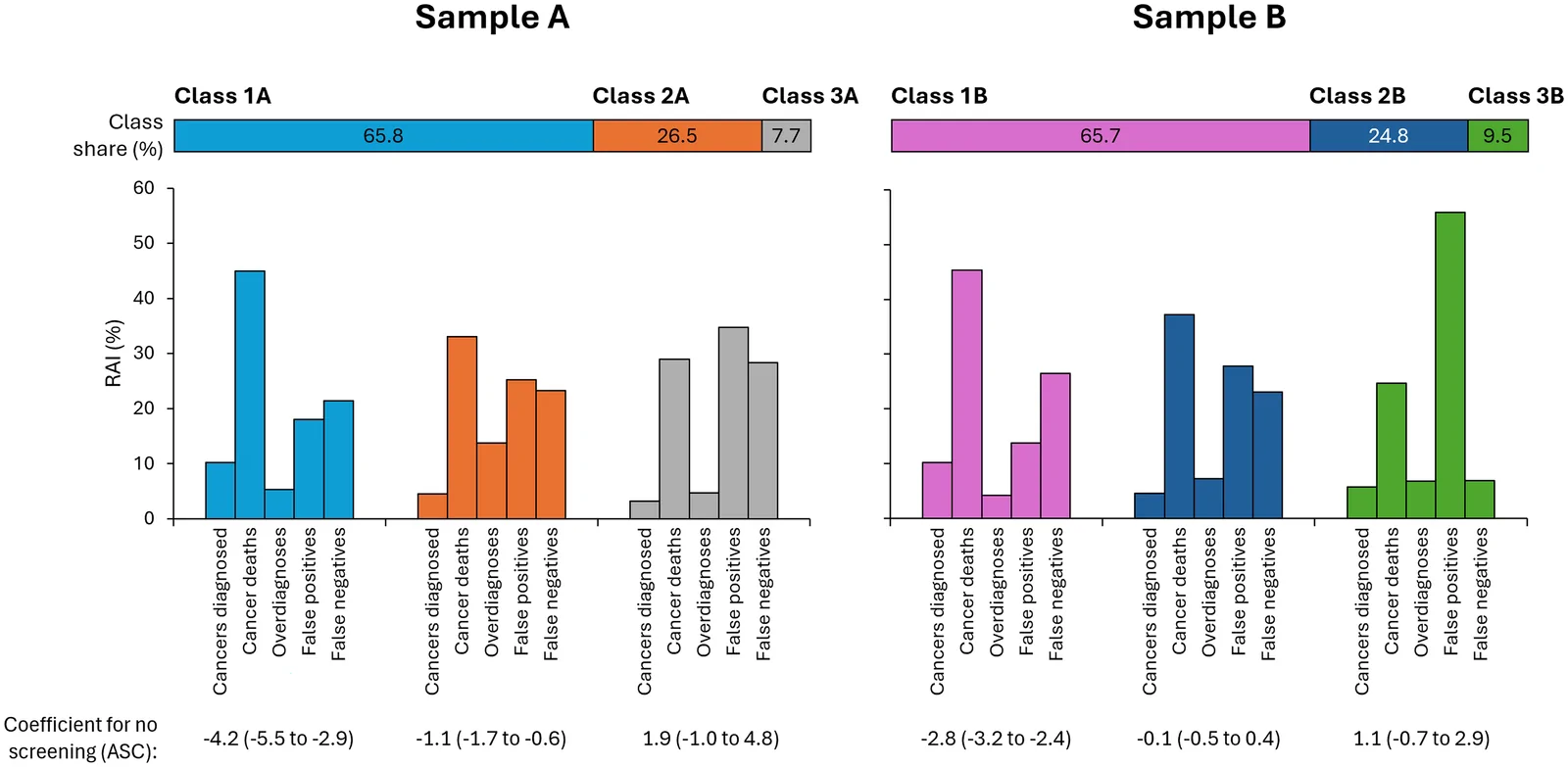
Relative attribute importance by latent class.

Full results of the latent class analysis are presented in Table S6. Figure 1 compares the RAI to the different classes. Across all classes, participants preferred screening with the greatest benefits and least harms. Two-thirds of each sample (Class 1A/B) comprised participants with the strongest preferences for screening (large negative coefficient for no screening) and who placed greatest importance on preventing deaths (RAI highest for cancer deaths). The second-largest class in Sample A (Class 2A; 27%) also favoured screening over no screening and prioritised preventing deaths, but tended to also value all the other attributes when making their selection. The second-largest class in Sample B (Class 2B, 25%) had a similar RAI distribution to that of Class 2A, but did not show an overall significant preference for/against screening. The remaining 8%–10% participants formed Class 3A/3B in which fewer attributes were statistically associated with the selected option than in the other classes. Of note, false positives were particularly important to Class 3B.

ASC: alternative specific constant.

As also shown in Table S6, Class 1A was less likely to have a degree-level education and more likely to consider themselves likely to develop cancer than the other classes. Class 1B was more likely to be aged over 50 years than Class 3B.

### Predicted probability of preferring screening programmes with different levels of harms

**Figure 2.**
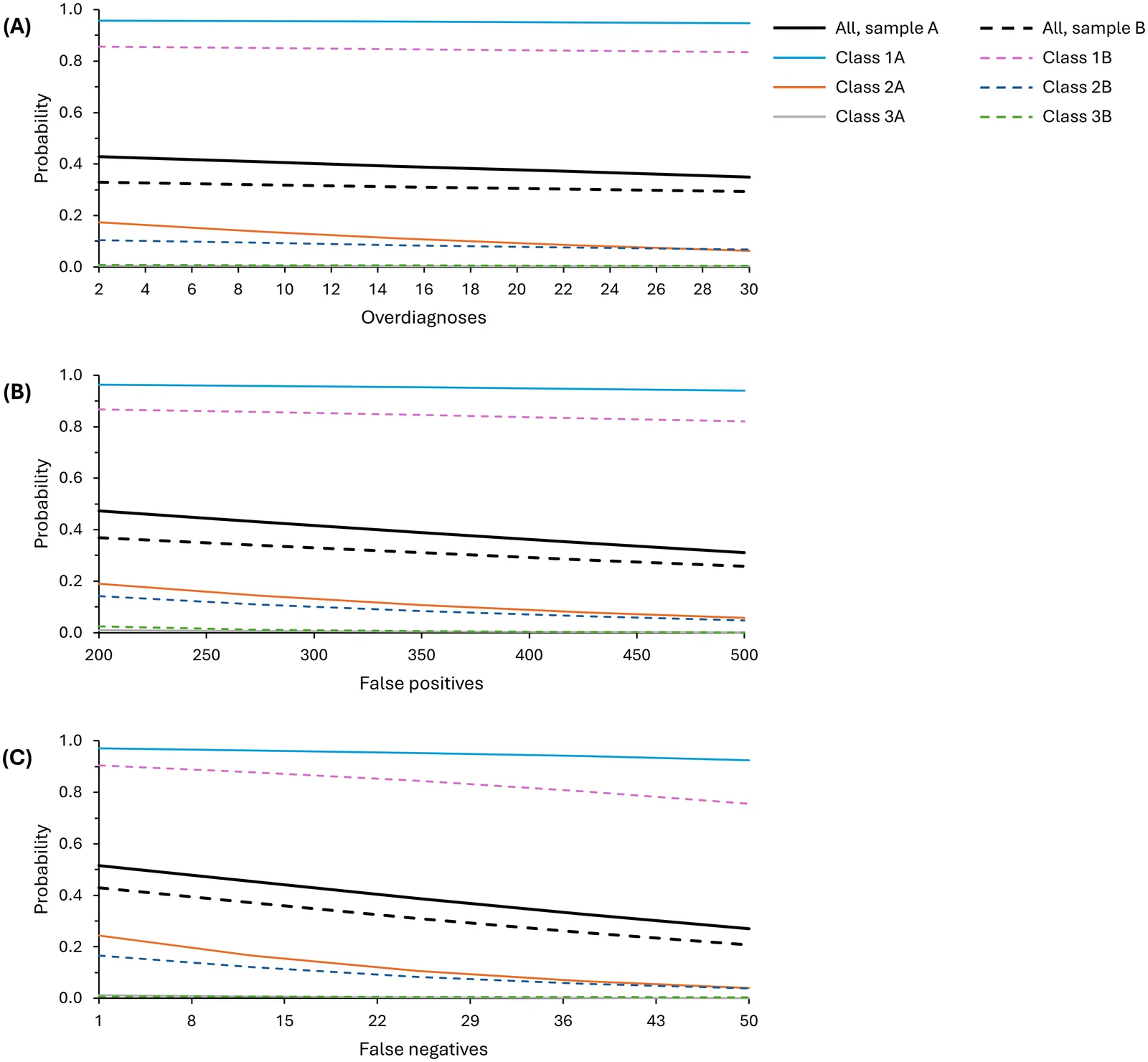
Modelled probability of preferring screening programmes with different levels of harms but no benefits compared to no screening. (A) Changing number of overdiagnoses, (B) changing number of false positives and (C) changing number of false negatives. In each case, the other harms were modelled as: 16 overdiagnoses, 350 false positives and 25 false negatives. No screening (comparator): no screening constant, 50 cancers diagnosed, 10 cancer deaths and 0 harms.

Figure 2 presents the probability that participants would prefer screening with varying harms in the absence of benefits (with zero deaths and cancers prevented), changing each harm in turn and keeping the other harms at the average levels, compared to no screening. Considering each sample as a whole, participants were unlikely to opt for these programmes over no screening with probability of preferring screening <0.5, that is, less than the probability for no screening. Taking the latent classes separately, however, Classes 1A and 1B would almost certainly prefer screening with no benefit over no screening (>92% and >76%, respectively), whereas all other classes would likely never prefer screening if there were no benefits over no screening.

Changing the level of one screening harm had small-to-medium impact on probability of preferring screening across the levels modelled. Changing the number of overdiagnoses had the smallest impact with a difference of 4%–8%, while changing the number of false negatives made a difference of 22%–25% probability of preference.

### Predicted probability of preferring screening comparable to current programmes

**Figure 3.**
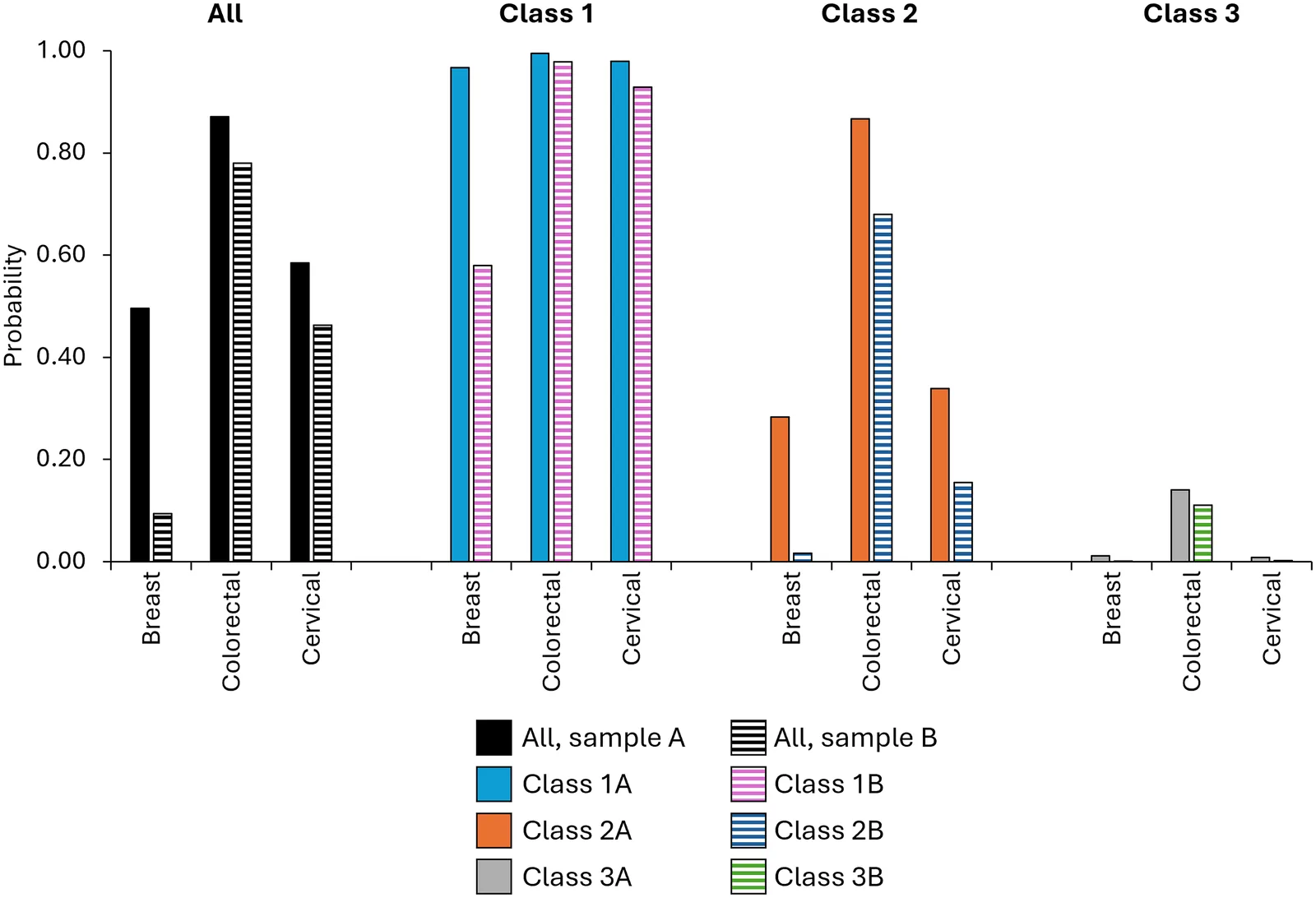
Modelled probability of preferring different screening programmes with benefits and harms comparable to current UK screening programmes, compared to no screening, for each type of cancer. Values used in each model are explained in Supplementary File 2, Table S1.

Figure 3 shows the probability that participants would select screening based on the different numbers of benefits and harms associated with current programmes, compared to no screening. Overall, programmes like colorectal screening where there is a reduction in both cancers diagnosed and cancer deaths would be likely to be taken up by the majority (78%–87% probability), while programmes like breast screening where screening results in an increase in cancers diagnosed would be least likely to be taken up (9%–50%). The probability of taking up screening was >50% for all screening programmes in Class 1, only for colorectal screening in Class 2, and not for any programmes in Class 3.

ASC: alternative specific constant.

### DCE evaluation

Most participants found the DCE difficult, although more in Sample A found it difficult than Sample B (80% versus 63% respectively, *p* < 0.001; Table S7). Nevertheless, strategies for completing the DCE were comparable (*p* = 0.064), with approximately half of each sample taking all attributes into account. Number of deaths prevented was utilised by 88% participants, cancers prevented and false negatives by two-thirds of participants, and overdiagnoses and false positives by half of participants. Again, no differences were seen between the samples. This is reflected in their ranking of the attributes (Figure S1): deaths prevented was most important, followed by cancers prevented then false negatives. This is somewhat consistent with the DCE RAI when participants were forced to make trade-offs.

## Discussion

This study adds to the growing literature on the positivity of the public towards cancer screening, indicating that harms do not carry significant weight with the majority when considering screening options. Specifically, approximately two-thirds would – hypothetically – choose screening over no screening at all ranges of benefit/harm including no benefit scenarios; those remaining would be more likely influenced by benefit/harm trade-offs. Programmes that reduce cancer mortality are preferred over those preventing cancer and minimising false positives and negatives is deemed more important than overdiagnosis level. As discussed below, these choices were made following information about the lifetime impacts of screening. This highlights the importance of screening communication targeted to these key viewpoints, as well as the need for policymakers and those delivering screening programmes to recognise that the majority of those invited to screening will accept without considering the specific benefits and harms to themselves. These considerations accentuate the onus on policymakers when appraising evidence or supporting the generation of new evidence to support public health decisions about the introduction or modification of cancer screening programmes.

Our findings reiterate the consistent support of the UK population, as a whole, for screening that has been seen over the past 15 years. This includes general enthusiasm about screening^4^ as well as positivity towards screening without beneficial cancer outcomes.^4,6,7^ In addition to the majority who would almost certainly take up screening with no benefits only harms, we also identified an important subgroup who support screening less strongly and for whom each outcome has weight. However, we were unable to identify sociodemographic characteristics that were consistently associated with these classes across both samples. A comparable distinction between most who will take up any screening versus a significant minority whose intentions vary by screening benefits and harms has been observed previously in the context of colorectal cancer screening decisions that vary by test modality and baseline risk level.^32^ The remaining participants in our study did not show such clear priorities for screening and may be considered as ‘other’, that is, they formed Class 3A/3B because they did not fit into the other classes. Furthermore, aside from the first showing stronger screening preferences, the distinct subgroups were similar across the sample with a specific healthcare interest and the general population sample, both in their proportion and views.

The finding that preventing deaths through screening is the most important attribute was unsurprising.^33^ For example, it had a RAI of 59% in a previous DCE about risk-stratified colorectal cancer screening, with participants explaining how it was hard to select against it.^6,34^ Elsewhere, participants have similarly indicated the overriding importance of the potential for just one person to benefit from screening and a lack of engagement with harms.^32^ Nevertheless, the low importance of cancer prevention, relative to mortality prevention, was striking. Although reasons for the relative importance of each harm are unclear, it perhaps reflects the pre-existing public knowledge of false positives and lack of understanding of overdiagnosis.^35^

Using coefficients from the full samples, our modelled estimates of colorectal cancer screening uptake based on the benefits and harms alone (78%–87%) were higher than the 72% observed in England in 2023/24.^36^ Conversely, our estimates of cervical screening uptake (46%–58%) were lower than the 68% observed, and our estimates of breast screening uptake (9%–50%) were substantially lower than the 70% observed.^36^ Our results are driven by lives saved through screening, which is proportionally highest for colorectal screening. Conversely, there are multiple factors at play in real decisions about screening attendance. These include attitudes towards healthcare services, the screening process, personal perspectives about risk, and social values of screening programmes,^3,37^ as well as differences between intention and enacted attendance. Additionally, this represents the views of all adult participants, not only those eligible for each screening programme, and some of the values modelled fell above the range of that presented in the DCE.

The modelled estimates of screening uptake may have resulted from the information we gave participants on screening outcomes because we sought to ensure that participants made their decisions based on the numbers of benefits and harms presented on the same scale: that which could be expected across the lifetime for people screened regularly. This could have been surprising to participants because although harms are included, screening communication tends to present long-term benefits and short-term harms, such as chance of a false positive at each mammography.^38^ This makes comparisons difficult and potentially inflates the benefits. Additionally, comprehensive information about screening harms can cause discomfort and result in the information being rejected where it goes against existing perceptions,^39–41^ as we observed with PPI representatives when developing the survey. Specifically, information about risk of false positive and negative cancer screening results has been associated with a more negative assessment of the theoretical decision to take up screening compared to when there is no information about harms.^40^ Nevertheless, we cannot comment on whether similar thoughts impacted participants’ decisions between screening options.

### Strengths and limitations

A strength of this study is the inclusion of information for participants to learn about lifetime screening harms, alongside the benefits, before they made their decisions. The background information was both described in moderate detail before the DCE, and participants could access a brief explanation of each attribute within each DCE question, alongside the words and visuals. Despite taking a median 15–23 min to complete the survey, we are unable to report whether the information was read, understood and utilised, and how well each choice set was understood. We also did not include any internal validity checks within the survey design. Consequently, findings such as false positives being more important than overdiagnoses could be explained by poor understanding of these concepts or inconsistent responses.

Additionally, the design of this DCE meant that participants’ decisions about screening options were based on numerical outcomes alone: other attributes such as reassurance from a negative test were not included. Although the levels for each attribute were based on outcomes for UK cancer screening programmes, the questions were posed in the context of an unnamed cancer with the specifics of the screening, diagnostic testing and treatment processes undescribed. This could have made the exercise seem more abstract, detracted from the gravity of a particular type of cancer and mitigated some of the social values of screening programmes as discussed above.

Another strength is that we collected and compared perspectives of a relatively large sample of the UK population, part those representative with regards to age, sex and ethnicity and part those with an interest in healthcare. However, the online setting relied on computer/internet access meaning some potential participants were excluded; this may include many poorly served by health services. Additionally, further selection bias inevitably remained in both samples, with people more willing to engage in the topic more likely to start and complete the survey.

### Implications

For most, providing explicit information about the harms of screening does not impact their overall choices even when no individual benefits are stated. Additionally, offering screening through the National Health Service can be inherently considered an endorsement of its individual and wider altruistic benefits.^42,43^ This consequently highlights the ongoing importance of ensuring screening programmes are only implemented when there is objective and robust evidence of benefits to the population as a whole.^44^ Nevertheless, addressing the information needs of groups with distinct perspectives towards cancer screening should not be overlooked. As risk-stratified screening (programmes without traditional sex and/or age-based criteria) and multicancer detection tests become more widely available and implemented, it will be important to justify differential screening schedules to the two-thirds of the population who are positively disposed towards cancer screening. This is particularly true when less screening is offered to individuals at low risk, who may be seen to miss out on potential screening benefits rather than have a reduced risk of harm.^45^ The sub-population who have a greater tendency to weigh-up both benefits and harms may be eager for more information with which to decide whether to attend screening.

Given the potentially conflicting aims of promoting screening participation and informed decision-making,^10^ targeting different groups within public health campaigns and screening information leaflets is not straightforward and will require careful development and evaluation. Making different levels of information available may be a worthwhile strategy. Additionally, it is vital to generate more detailed evidence on who is likely to make up the population sub-groups based on sociodemographics and/or other harder to measure characteristics to inform and evaluate targeted communication.

## Conclusions

Through a DCE, this study highlights the public's preferences for cancer screening, with decisions between screening options driven by the number of deaths that will be prevented above other benefits and harms. Furthermore, it suggests that approximately two-thirds of the population would opt for cancer screening without direct personal benefit in terms of incidence and mortality. These findings emphasise the importance of only implementing programmes with net benefit for the eligible population as indicated by robust assessment of existing, or the generation of additional, evidence.

## Supplemental Material

sj-pdf-1-msc-10.1177_09691413261422933 - Supplemental material for Public (un)willingness to trade-off benefits and harms in decisions about cancer screening: A discrete choice experiment Supplemental material, sj-pdf-1-msc-10.1177_09691413261422933 for Public (un)willingness to trade-off benefits and harms in decisions about cancer screening: A discrete choice experiment by Rebecca A Dennison, Stuart Wright, Georgios Lyratzopoulos, Nora Pashayan, Stephen Morris, Brian Nicholson, and Juliet A. Usher-Smith in Journal of Medical Screening

sj-pdf-2-msc-10.1177_09691413261422933 - Supplemental material for Public (un)willingness to trade-off benefits and harms in decisions about cancer screening: A discrete choice experiment Supplemental material, sj-pdf-2-msc-10.1177_09691413261422933 for Public (un)willingness to trade-off benefits and harms in decisions about cancer screening: A discrete choice experiment by Rebecca A Dennison, Stuart Wright, Georgios Lyratzopoulos, Nora Pashayan, Stephen Morris, Brian Nicholson, and Juliet A. Usher-Smith in Journal of Medical Screening
