# Supplementary material for: Public (un)willingness to trade-off benefits and harms in decisions about cancer screening: A discrete choice experiment
Source: J Med Screen. 2026 Mar 5;33(3):152–62. doi: 10.1177/09691413261422933 (PMC13490661; doi:10.1177/09691413261422933)
Supplement: Supplementary file 1 [file sj-pdf-1-msc-10.1177_09691413261422933.pdf]

Public (un)willingness to trade-off benefits and harms in decisions about cancer screening: A discrete choice experiment

Supplementary file 1: Survey outline

[Text in blue was not shown to participants. Grey boxes indicate questions posed]

Part 1. Beliefs about cancer screening (1)

Your thoughts about cancer screening

Thank you for agreeing to take part in this research.

Before starting the main part of the survey, we want to understand your thoughts and beliefs about cancer screening. Please select the response that best describes your view.

Do you think routine cancer screening tests for healthy people are almost always a good idea?	• Yes<br>• No<br>• Don't know
How often does finding cancer early mean that treatment saves lives?	• None of the time<br>• Some of the time<br>• Most of the time<br>• All the time<br>• Don't know
How often does finding cancer early mean that a person can have less treatment?	• None of the time<br>• Some of the time<br>• Most of the time<br>• All the time<br>• Don't know
Have you ever heard of cancers that grow so slowly that they are unlikely to cause you any problems in your lifetime?	• Yes<br>• No<br>• Don't know
Would you want to be tested to see if you had a slow-growing cancer like that?	• Yes<br>• No<br>• Don't know
If there was a kind of cancer for which nothing could be done, would you want to be tested to see if you had it?	• Yes<br>• No<br>• Don't know
In the past, do you think you have had too many screening tests for cancer, too few or about the right number?	• Too few<br>• About right<br>• Too many<br>• Don't know
Do you feel that someone who does not go for screening is irresponsible?	• Yes<br>• No<br>• Don't know

<next page>

Part 2. Conjoint-analysis tasks

1. Explanation of each of the attributes and levels

Instructions

We will start by explaining the ideas included in the questions. Don't worry if you don't remember the information because the key points will be repeated on the question pages.

<next page>

Information about cancer screening

Screening involves an individual with no sign or symptoms of cancer doing a screening test.

If the test finds something or suggests that the person may be at higher risk of developing a health problem, the person is then offered more tests to investigate.

Examples of cancer screening programmes offered in the UK are shown in the table.

Type of cancer	Who is offered screening	Screening test	Possible tests used for further investigations
Bowel[1]	Men and women aged 50 (or 60 depending on region) to 74	Home test kit: this is used to collect a small sample of poo and send it to a lab. This is checked for tiny amounts of blood	Colonoscopy: a thin tube with a camera inside is passed into your bottom to look for signs of bowel cancer
Breast[2]	Women aged 50-71	Mammogram: X-rays of the breasts	An examination of the breast<br>More mammograms<br>Ultrasound scans of the breast<br>Biopsy: taking a small sample from the breast using a needle
Cervical[3]	Women aged 25-64	Smear test: a small sample of cells are taken from the cervix (the opening to the womb from the vagina) to check for certain types of human papillomavirus (HPV) that can cause changes to the cells of the cervix	Another smear test at a later date<br>Colposcopy: a different test to look at the cervix and possible biopsy

Find out more about each screening option *<option to show/hide shaded text>*

This is copied from the information provided by the NHS on each screening programme:

1 https://www.nhs.uk/conditions/bowel-cancer-screening

2 https://www.nhs.uk/conditions/breast-screening-mammogram

3 https://www.nhs.uk/conditions/cervical-screening

<next page>

Benefits and harms of cancer screening

The NHS aims to offer screening only when the benefits outweigh the harms. While the screening offered through the NHS has been shown to be beneficial for improving health outcomes across the country and allowing for informed decision-making about health, for each individual there are both benefits and harms.

We would like you to consider five benefits and harms when completing the questions about cancer screening. **You will be able to see information about these alongside each of the questions.**

1. Screening can **prevent deaths from the specific cancer** through earlier diagnosis and treatment.
2. Screening can **prevent the specific cancer** by finding and treating cell abnormalities before cancer develops. This treatment is likely to be a one-off and may take place during the investigation and not require future appointments.
3. Screening can lead to **overdetection and overtreatment**. Some people will be diagnosed with a cancer that would not have made them ill during their lifetime and they have treatment they didn't need. They wouldn't know this but would think that screening had saved their life.
4. Some people need further investigations and these show that they do not have cancer. These are known as **false positives**: the screening test was wrong and they didn't actually need to have extra tests that might have been invasive.
5. Other people receive a negative screening test result when they actually do have cancer. These are known as **false negatives**: they are falsely reassured, which may delay diagnosis and treatment or cause them to dismiss symptoms.

Note, there are also other benefits and harms not included in this list. We have included ones that it is possible to count or estimate.

Overdetection, false negatives and false positives can have effects on physical and mental health, financial strain, and opportunity costs explained below. Unlike the benefits, these might be less obvious to people

Extra information about harms *<option to show/hide shaded text>*

- Further investigations could be invasive and cause pain (e.g., if a biopsy is taken) and sometimes complications arise (e.g., colonoscopy to look for signs of bowel cancer has a small risk of complications such as bleeding, injury to the bowel requiring hospitalisation and, very rarely, death)*.
- Treatment for cancer, which can include surgery, radiotherapy, chemotherapy and hormone therapy.
- Mental health impact of a potential or actual cancer diagnosis. This can include anxiety, loss of identity, and changing thoughts about the future for both the person screened and their friends and family.
- Costs associated with attending appointments and possible impact on insurance premiums.
- Needing to take time off work and usual activities for appointments and treatment.

* Risk of bleeding: 24 per 10,000 colonoscopies, risk of bowel perforation: 6 per 10,000 colonoscopies, and risk of death: 0.3 per 10,000 colonoscopies (Kothari et al. 2019 https://doi.org/10.1016/j.gie.2019.07.033).

<next page>

Context

In the following questions, we will present the benefits and harms of screening for a population of 1,000 people who do and do not undergo screening for a particular cancer:

- *No screening* means that people will **never** have screening for this cancer.
- *Screening* means that people complete a screening test **on a regular basis** (once every 2 to 3 years) **over a period of 20 years**.
- We refer to screening benefits and harms over people's **lifetime**, not just one test. These results may seem high because sometimes they are described for one-off tests that do not account for a person's lifetime.
- The numbers of benefits and harms shown are based on the range that might be expected from current cancer screening programmes in the UK.

<next page>

2. Conjoint-analysis task/DCE

Let's get started!

We will present you with a set of nine questions with two different screening options. Each time the number of benefits and harms will change.

For each question, please select the answer that you would choose if you were invited to screening. These questions are mandatory, so you won't be able to skip any of them.

Sometimes the answer might seem obvious to you. Other times it might be harder to work out what you think is best. **The questions are about your opinion so there is not a correct answer.**

<next page>

Question 1 of 9 [example 1]

This shows what might happen, on average, to 1,000 people who do and do not have screening on a regular basis over a period of 20 years. **Select the red question marks** for an explanation of each category.

**No screening**		
Out of 1,000 people without screening:<br>• **50 people** will be diagnosed with the cancer.<br>• **10 people** will die from the cancer.<br>(and 0 will have overdetection and overtreatment, false positives, or false negatives)		
**Category**	**Screening option 1**	**Screening option 2**
Diagnosis and cancers prevented ?	• **49 people** will be diagnosed with the cancer.<br>**1 cancer** will be prevented through screening.	• **48 people** will be diagnosed with the cancer.<br>**2 cancers** will be prevented through screening.
Deaths and deaths prevented ?	• **5 people** will die from the cancer.<br>**5 cancer deaths** will be prevented through screening.	• **10 people** will die from the cancer.<br>**0 cancer deaths** will be prevented through screening.
Overdetection and overtreatment ?	• **30 people** will be diagnosed but the cancer would not have caused them harm or have killed them.	• **15 people** will be diagnosed but the cancer would not have caused them harm or have killed them.
False positives ?	• **200 people** will have a positive screening test but further investigations show they do not have cancer.	• **300 people** will have a positive screening test but further investigations show they do not have cancer.
False negatives ?	• **10 people** will have a negative screening test when they actually do have cancer.	• **20 people** will have a negative screening test when they actually do have cancer.

Show/hide this visual ✖

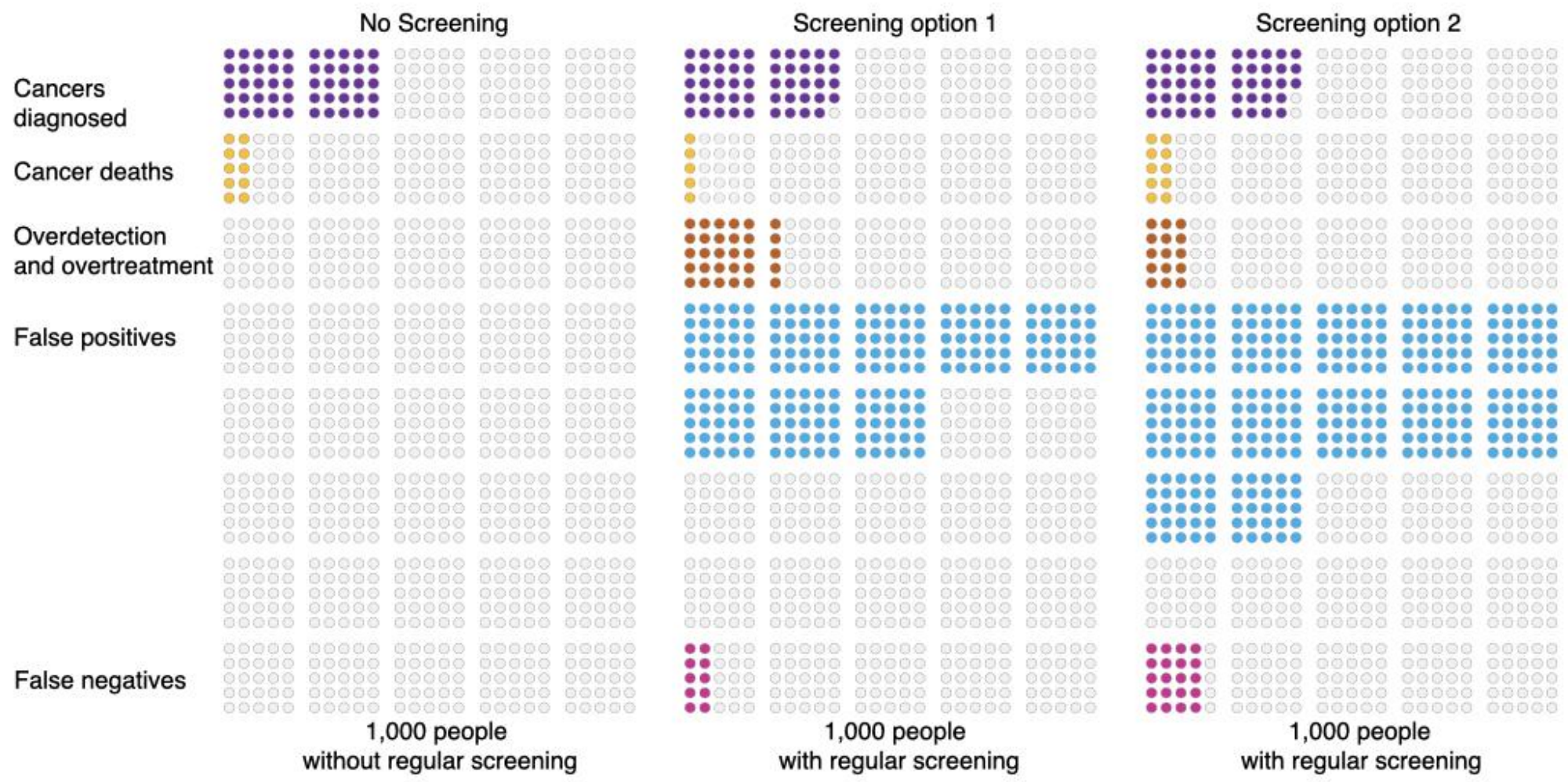

Imagine that you were invited to take part in screening for this cancer. Which option would you choose?

○ **No screening** ○ **Screening option 1** ○ **Screening option 2**

<When participants hover over the question marks, the following text will appear>

Cancers prevented

This shows the number of people who will **not develop cancer** because it is **prevented** by treating abnormalities detected by screening before they develop into cancer.

Deaths prevented

This shows the number of **deaths from this cancer** that will be **prevented** through earlier diagnosis and treatment.

Overdetection and overtreatment

This shows the number of people who will be diagnosed with a **cancer that would not have made them ill or have killed them** (and they wouldn't know this).
The effects of this may include:

- Surgery and other cancer treatment
- Anxiety and loss of identity due to a cancer diagnosis
- Having to take time off work and usual activities for treatment
- Costs associated with attending appointments

False positives

This shows the number of people who receive a **positive screening test result** but further investigations show that they **do not have cancer**. The screening test was wrong, meaning they didn't actually need to have further investigations that might have been invasive like a biopsy or colonoscopy.
The effects of this may include:

- Pain and risk of hospitalisation due to complications from further investigations
- Anxiety and loss of identity due to a possible cancer diagnosis
- Having to take time off work and usual activities at attend appointments
- Costs associated with attending appointments or increased insurance

False negatives

This shows the number of people who will be **falsely reassured** because they have a negative screening test when they actually **do have cancer**.
The effects of this may include:

- Delays to diagnosis and treatment
- Dismissing symptoms if they arise
- Being less motivated to have a healthy lifestyle

<next page>

Part 3. DCE evaluation

Evaluation

Please tell us how you found the exercise.

In general, how easy or difficult did you find choosing between the different options?	• Very easy<br>• Easy<br>• Slightly easy<br>• Slightly difficult<br>• Difficult<br>• Very difficult
Please tell us a few words why you found choosing between the different programmes easy or difficult.	• [Free text]
Did you find yourself making choices based on one or two outcomes from the screening programme rather than all of them?	• Yes - I based choices on one or two outcomes<br>• No - I considered all or nearly all the outcomes
Which outcomes did you use to help you choose between the options? If you used all the outcomes, please select them all.	• Number of deaths from cancer that will be prevented<br>• Number of cancers that will be prevented<br>• Number of people diagnosed with a cancer that would not have caused them harm<br>• Number of people with a positive screening test but further investigations show that they do not have cancer<br>• Number of people with a negative screening test when they actually do have cancer
Please rank the outcomes in order of their importance to you when choosing between the different options. 1 (most important) – 5 (least important)	• Number of deaths from cancer that will be prevented<br>• Number of cancers that will be prevented<br>• Number of people diagnosed with a cancer that would not have caused them harm<br>• Number of people with a positive screening test but further investigations show that they do not have cancer<br>• Number of people with a negative screening test when they actually do have cancer

<next page>

Part 4. Demographics and cancer worry (2–4)

About you

In this last section, please answer a few questions about yourself. These questions allow us to make sure we include people from a range of backgrounds and see if different groups of people have different views. We will not be able to identify you from your answers.

How old are you?	• ___ years<br>• Prefer not to say
What is your biological sex? (A question about gender identity follows)	• Female<br>• Male<br>• Prefer not to say
Is the gender you identify with the same as your sex registered at birth?	• Yes<br>• No (please enter your gender identity)<br>• Prefer not to say
Which option best describes your ethnic group or background?	• White<br>o English, Welsh, Scottish, Northern Irish or British<br>o Irish<br>o Gypsy or Irish Traveller<br>o Roma<br>o Any other White background, please describe_________<br>• Mixed or Multiple ethnic groups<br>o White and Black Caribbean<br>o White and Black African<br>o White and Asian<br>o Any other Mixed or Multiple ethnic background, please describe_________<br>• Asian or Asian British<br>o Indian<br>o Pakistani<br>o Bangladeshi<br>o Chinese<br>o Any other Asian background, please describe_________<br>• Black, Black British, Caribbean or African<br>o Caribbean<br>o African, please describe_________<br>o Any other Black, Black British or Caribbean background, please describe_________<br>• Other ethnic group<br>o Arab<br>o Any other ethnic group, please describe_________<br>• Prefer not to say
What is your highest education level?	• Finished school at or before the age of 15 years<br>• Completed GCSEs, O Levels or equivalent<br>• Completed A Levels or equivalent<br>• Completed further education but not a degree<br>• Completed a Bachelor's degree

	• Completed a Master's degree, PhD or other postgraduate qualification<br>• Other (please describe)
Where would you put yourself on the socio-economic ladder? *<br><br>*Think of this ladder as representing where people stand in our society. At the top of the ladder (10) are people better off with most education, money and desirable jobs. At the bottom of the ladder (1) are people worse off with least education, money and undesirable jobs.	• 10 – 1<br>• Prefer not to say
Have you ever had cancer?	• Yes<br>• No<br>• Prefer not to say
Have your parents or any brothers or sisters ever had cancer?	• Yes<br>• No<br>• Don't know<br>• Prefer not to say
Has anyone close to you (e.g. a partner or close friend) ever had cancer?	• Yes<br>• No<br>• Don't know<br>• Prefer not to say
Have you ever taken part in a cancer screening programme? Examples include:<br><br>Bowel cancer screening (poo sample, men and women aged 50 (or 60 depending on region) to 74)<br><br>Breast cancer screening (mammogram, women aged 50-71)<br><br>Cervical cancer screening (smear test, women aged 25-64)	• Yes<br>• No – I have chosen not to have screening<br>• No – I haven't been invited to have screening<br>• Prefer not to say
How likely do you think is it that you will get cancer at some point in the next 10 years?	• Extremely likely<br>• Moderately likely<br>• Slightly likely<br>• Neither likely nor unlikely<br>• Slightly unlikely<br>• Moderately unlikely<br>• Extremely unlikely<br>• Prefer not to say

During the past month, how often have you thought about your own chances of getting cancer?	• Not at all<br>• Rarely<br>• Sometimes<br>• Often<br>• A lot<br>• Prefer not to say

References

1. Waller J, Osborne K, Wardle J. Enthusiasm for cancer screening in Great Britain: a general population survey. Br J Cancer. 2015 Feb 3;112(3):562–6.
2. Company PAM. PAMCo Interview and questionnaire [Internet]. [cited 2021 Aug 9]. Available from: https://pamco.co.uk/how-it-all-works/interview-and-questionnaire/
3. Cancer Research UK, University College London, Kings College London and U of O. Cancer Awareness Measure toolkit version 2.1 Cancer Research UK Cancer Awareness Measure (CAM) Toolkit (version 2.1) [Internet]. 2007. Available from: https://www.cancerresearchuk.org/sites/default/files/health_professional_cancer_awareness_measure_toolkit_version_2.1_09.02.11.pdf
4. Lerman C, Trock B, Rimer BK, Boyce A, Jepson C, Engstrom PF. Psychological and behavioral implications of abnormal mammograms. Ann Intern Med. 1991 Apr;114(8):657–61.
