# Supplementary material for: Public (un)willingness to trade-off benefits and harms in decisions about cancer screening: A discrete choice experiment
Source: J Med Screen. 2026 Mar 5;33(3):152–62. doi: 10.1177/09691413261422933 (PMC13490661; doi:10.1177/09691413261422933)
Supplement: Supplementary file 2 [file sj-pdf-2-msc-10.1177_09691413261422933.pdf]

Public (un)willingness to trade-off benefits and harms in decisions about cancer screening: A discrete choice experiment

Supplementary file 2: Supplementary tables and figures

Table S1. Additional details of the statistical analysis.

**1. Investigating scale and preference heterogeneity**<br>In order to inform whether Samples A and B and Versions 1 and 2 could be combined or should be analysed separately, we investigated potential scale heterogeneity (the degree to which randomness/error varies across respondents) and preference heterogeneity (the degree to which preferences vary across respondents) using Swait and Louviere plots [1,2].
**2. Selecting the logistic regression model**<br>The model that best fit the dataset was selected through examination of the Akaike and Bayesian Information Criteria (AIC and BIC). Optimal model specification was chosen using BIC.<br>First, basic linear conditional fixed-effects logistic regression models were estimated using continuous attributes, with and without constants for options 1 and 2.<br>Second, we assessed non-linearity within the attributes by effects-coding each attribute in turn.<br>For any attribute where model fit was better when included as a categorical variable than continuous variable, quadratic, logarithmic and piecewise transformations were performed.
**3. Calculating relative attribute importance (RAI)**<br>For each model, the relative importance of each attribute was calculated using attribute-based normalisation [3]. This was the difference in the coefficients multiplied by the most and least preferred level, presented as a percentage.
**4. Latent class analysis (LCA)**<br>LCA was used to investigate preference heterogeneity (i.e., to identify different groups within the population that hold differing views) and whether the resulting classes could be predicted by participant characteristics. The optimum number of classes, starting seed, and characteristics to describe class membership were determined by examining the BIC and considering the value added by more complex models.<br>The following binary characteristics to describe class membership were examined:<br>Age (older versus younger than 50 years)<br>Sex (female versus male)<br>Ethnicity (white versus any other ethnicity)<br>Education (degree-level education or above versus no degree)<br>Perceived position on the socioeconomic ladder (1-3 (low) versus 4-10 (high))<br>Prior cancer diagnosis (personal cancer history versus no cancer history or not reported)<br>Cancer screening participation experience (previously screened versus not had screening (declined, not eligible or not reported))<br>Self-reported assessment of likelihood to develop cancer in the next 10 years (likely versus unlikely or unsure)<br>Worry about developing cancer (frequently worried versus not worried)

5. Probability screening programmes with different levels of benefits and harms would be taken up

Lastly, we calculated the probability that the public would take up screening programmes with different levels of benefits and harms.

First, we modelled uptake of screening without anticipated benefits but different levels of harms: each harm (false positives, false negatives and overdiagnoses) was modelled between the lowest and highest level in turn, with the other two harms set to their average levels. No benefits were included (zero deaths prevented and zero cancer diagnoses prevented).

Second, we modelled uptake with lifetime screening benefits and harms comparable to current UK cancer screening programmes, compared to if there was no screening programme for each cancer type. The values used in the model are explained in Table S2 below.

For both analyses, no screening was used as the comparator. This was modelled as the no screening constant, 50 cancers diagnosed, 10 cancer deaths and zero harms.

Probability of uptake was calculated for both the samples as a whole and each latent class.

Table S1 references

[1] Wright SJ, Vass CM, Sim G, Burton M, Fiebig DG, Payne K. Accounting for scale heterogeneity in healthcare-related discrete choice experiments when comparing stated preferences: A systematic review. Patient. 2018;11(5):475-488. doi: 10.1007/s40271-018-0304-x.

[2] Swait J, Louviere J. The role of the scale parameter in the estimation and comparison of multinomial logit models. J. Mark. Res. 1993;30:305–14. doi: 10.2307/3172883.

[3] Hauber AB, González JM, Groothuis-Oudshoorn CGM, et al. Statistical methods for the analysis of discrete choice experiments: A report of the ISPOR conjoint analysis good research practices task force. Value in Health 2016; 19: 300–315. doi: 10.1016/j.jval.2016.04.004.

Table S2. Values used to model the probability of preferring different screening programmes with benefits and harms comparable to current UK screening programmes, compared to no screening for each cancer (Figure 4).

	No screening		With screening	
	**Value (per 1,000)**	**Justification/reference**	**Value (per 1,000)**	**Justification/reference**
**Breast**				
**Cancers diagnosed**	55.2	Calculated as difference between cancers diagnosed with screening and overdiagnosis (Also 18% of screen detected cancers are excess cancers [1], calculated from cancers diagnosed with screening)	68.1	For 10,000 women invited to screening, from age 50 for 20 years, an estimated 681 cancers (invasive and DCIS) will be diagnosed [2]
**Cancer deaths**	18.8	Invitation to breast screening is associated with an estimated 20% reduction in breast cancer mortality [2], calculated from cancer deaths with screening	15.0	10-year breast cancer survival of 78% [3], calculated from cancers diagnosed with screening
**Overdiagnoses**	0	No screening	12.9	19% of breast cancer diagnoses are estimated to represent overdiagnosis [2], calculated from cancers diagnosed with screening
**False positives**	0	No screening	464.0	464/1,000 women who have breast screening for 20 years experience a false positive result [4]
**False negatives**	0	No screening	4.2	Screening mammography has a false-negative rate of 0.7 per 1,000 screens [5], calculated as 6 screens over lifetime
**Colorectal**				
**Cancers diagnosed**	30.0	Incidence of 30/1,000 at 3% risk with no screening [6]	29.0	Incidence of 29/1,000 at 3% risk with 2-yearly FIT [6]
**Cancer deaths**	9.0	Mortality of 9/1,000 at 3% risk with no screening [6]	4.0	Mortality of 4/1,000 at 3% risk with 2-yearly FIT [6]
**Overdiagnoses**	0	No screening	2.9	Overdiagnosis for invasive CRCs, including high grade dysplasia, resulting from FIT screening is 9.9% [7], calculated from cancers diagnosed with screening
**False positives**	0	No screening	235.0	2-yearly FIT associated with 264 colonoscopies/1,000 people [6], calculated with cancers diagnosed with screening ('appropriate' colonoscopies)
**False negatives**	0	No screening	6.1	Pooled sensitivity of FIT for CRC was approximately 79% [8], calculated from cancers diagnosed with screening
**Cervical**				
**Cancers diagnosed**	7.7	Estimated lifetime risk of being diagnosed with cervical cancer is 1 in 130 [9]	4.9	36.9% of cervical cancer could be prevented by HPV screening [10], calculated from cancers diagnosed with no screening
**Cancer deaths**	2.8	10-year cervical cancer survival is 63% [11], calculated from 7.7/1,000 cancers diagnosed with no screening	1.8	10-year cervical cancer survival is 63% [11], calculated from cancers diagnosed with screening

**Overdiagnoses**	0	No screening	0.4	Frequency of overdiagnosis is 39.6/100,000 for HPV testing [12]
**False positives**	0	No screening	530.0	53.5% lifetime chance of a false positive cervical screen [13]
**False negatives**	0	No screening	0.3	HPV screening would have missed 4.3% observed cancers [10], calculated from cancers diagnosed without screening

Table S2 references

[1] Autier P, Ould Ammar R, Bota M. The influence of breast screening on breast cancer incidence in England: observational study based on cancer registries and bulletins of the NHS Breast Screening Programme. Eur J Public Health 2024;34:812–7. https://doi.org/10.1093/eurpub/ckae069.
[2] Marmot M, Altman D, Cameron D, Dewar J, Thompson S, Wilcox M, et al. The benefits and harms of breast cancer screening: an independent review. The Lancet 2012;380:1778–86. https://doi.org/10.1016/S0140-6736(12)61611-0.
[3] Cancer Research UK. Breast cancer survival statistics. Available from: https://www.cancerresearchuk.org/health-professional/cancer-statistics/statistics-by-cancer-type/breast-cancer/survival. Date accessed: August 2025.
[4] Stiggelbout A, Copp T, Jacklyn G, Jansen J, Liefers G-J, McCaffery K, et al. Women's Acceptance of Overdetection in Breast Cancer Screening: Can We Assess Harm-Benefit Tradeoffs? Medical Decision Making 2020;40:42–51. https://doi.org/10.1177/0272989X19886886.
[5] Durand MA, Friedewald SM, Plecha DM, Copit DS, Barke LD, Rose SL, et al. False-negative rates of breast cancer screening with and without digital breast tomosynthesis. Radiology 2021;298:296–305. https://doi.org/10.1148/radiol.2020202858.
[6] Helsingen LM, Vandvik PO, Jodal HC, Agoritsas T, Lytvyn L, Anderson JC, et al. Colorectal cancer screening with faecal immunochemical testing, sigmoidoscopy or colonoscopy: a clinical practice guideline. BMJ 2019:l5515. https://doi.org/10.1136/bmj.l5515.
[7] Lin T-Y, Chiu SY-H, Liao L-C, Chen SL-S, Chiu H-M, Chen TH-H. Assessing overdiagnosis of fecal immunological test screening for colorectal cancer with a digital twin approach. NPJ Digit Med 2023;6:24. https://doi.org/10.1038/s41746-023-00763-5.
[8] Lee JK, Liles EG, Bent S, Levin TR, Corley DA. Accuracy of fecal immunochemical tests for colorectal cancer. Ann Intern Med 2014;160:171–81. https://doi.org/10.7326/M13-1484.
[9] Cancer Research UK. Cervical cancer risk. Available from: https://www.cancerresearchuk.org/health-professional/cancer-statistics/statistics-by-cancer-type/cervical-cancer/risk-factors. Date accessed: August 2025.
[10] Castanon A, Landy R, Sasieni P. How much could primary human papillomavirus testing reduce cervical cancer incidence and morbidity? J Med Screen 2013;20:99–103. https://doi.org/10.1177/0969141313492313.
[11] Cancer Research UK. Cervical cancer survival statistics. Available from: https://www.cancerresearchuk.org/health-professional/cancer-statistics/statistics-by-cancer-type/cervical-cancer/survival. Date accessed: August 2025.
[12] Hamashima C, Hearasawa T, Katayama T, Sasaaki S, Hosono S, Hoshi K. 92 Systematic review of overdiagnosis in cervical cancer screening: how should we define overdiagnosis in cervical cancer screening? BMJ Evid Based Med, BMJ Publishing Group Ltd; 2018, p. A43.2-A44. https://doi.org/10.1136/bmjebm-2018-111070.92.
[13] White T, Algeri S. Estimating the lifetime risk of a false positive screening test result. PLoS One 2023;18:e0281153. https://doi.org/10.1371/journal.pone.0281153.

Table S3. Determining whether there are differences between Samples A and B, and Sample B Versions 1 and 2.

Swait and Louviere plot	R^2 to examine preference heterogeneity	Coefficient to examine scale heterogeneity	Notes
**A. Sample A (Thiscovery) versus Sample B (Prolific)**			
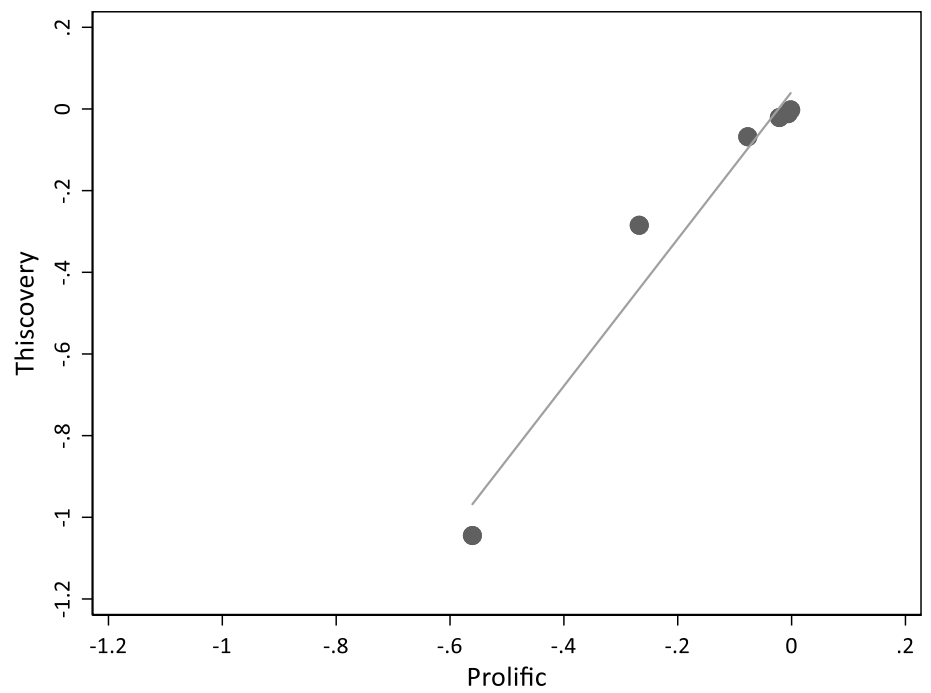	0.958<br><br>$R^2 \approx 1$ indicating no heterogeneity in preferences between the samples	1.801<br><br>Coefficient $\not\approx 1$ indicating scale heterogeneity (differences in error variance between the samples)	Analyse Sample A and B separately
**B. Sample A Version 1 versus Version 2**			
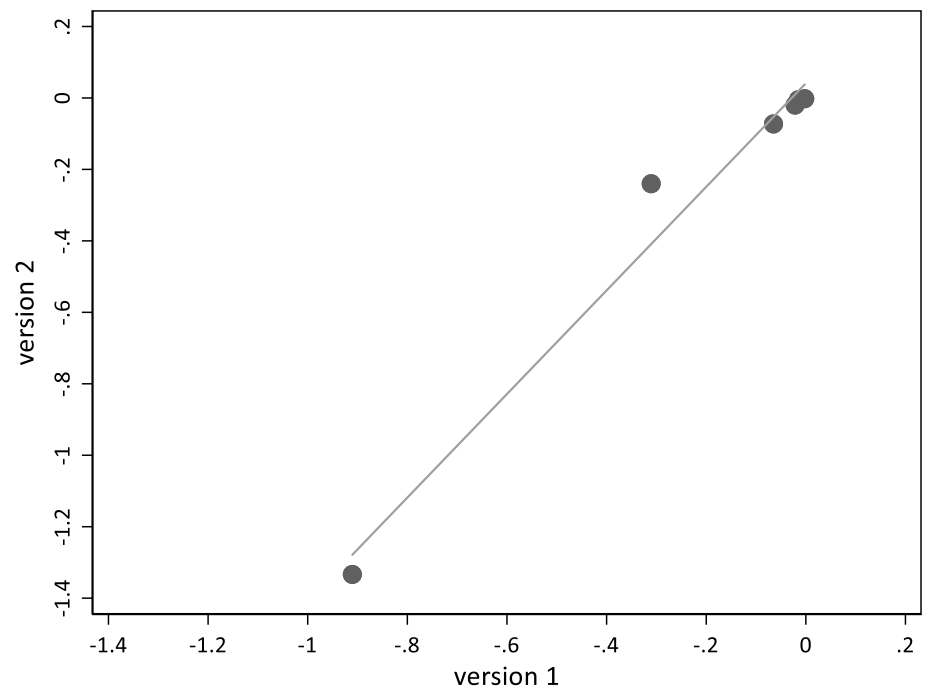	0.968<br><br>$R^2 \approx 1$ indicating no heterogeneity in preferences between the samples	1.450<br><br>Coefficient $\not\approx 1$ indicating scale heterogeneity (differences in error variance between the samples)	Use heteroscedastic conditional logit for Sample A

Table S4. Model selection for Samples A and B.

A. Selecting the best model to analyse Sample A.

	Model	LL	DF	AIC	BIC (n=414)	Notes
**Basic model**						
1	Basic heteroscedastic model (continuous coding of attributes; including constant for asc)	-3089.521	7	6193.042	6221.223	Best model
**Adding constants for preference for option 1 or 2 (to consider heuristics preferences)**						
2a	Model 1 plus constant for option 1	-3089.152	8	6194.304	6226.511	No improvement in model fit versus Model 1
2b	Model 1 plus constant for option 2	-3089.152	8	6194.304	6226.511	No improvement in model fit versus Model 1
**Effects coding each attribute in turn (to check for non-linearity)**						
3a	Model 1 with *cancers diagnosed* effects coded	-3085.063	9	6188.126	6224.359	No improvement in model fit versus Model 1
3b	Model 1 with *cancer deaths* effects coded	-3088.674	9	6195.349	6231.582	No improvement in model fit versus Model 1
3c	Model 1 with *overdiagnosis* effects coded	-3089.474	10	6198.947	6239.206	No improvement in model fit versus Model 1
3d	Model 1 with *false positives* effects coded	-3085.051	10	6190.103	6230.361	No improvement in model fit versus Model 1
3e	Model 1 with *false negatives* effects coded	NA				Model does not converge
**Best transformation for non-linear attributes**						
4	NA					

414 participants; 11,178 observations.

AIC: Akaike information criteria; asc: alternative specific constant; BIC: Bayesian information criteria (adjusted for sample size); DF: Degrees of freedom; NA: not applicable; LL: Log likelihood.

B. Selecting the best model to analyse Sample B.

	Model description	LL	DF	AIC	BIC (n=604)	Notes
**Basic model**						
1	Basic clogit model (continuous coding of attributes; including constant for asc)	-4797.354	6	9606.708	9633.129	
**Adding constants for preference for option 1 or 2 (to consider heuristics preferences)**						
2a	Model 1 plus constant for option 1	-4793.834	7	9601.669	9632.494	No substantial improvement in model fit versus Model 1
2b	Model 1 plus constant for option 2	-4793.834	7	9601.669	9632.494	No substantial improvement in model fit versus Model 1
**Effects coding each attribute in turn (to check for non-linearity)**						
3a	Model 1 with *cancers diagnosed* effects coded	-4785.122	8	9586.244	9621.473	Improved model fit versus Model 1 so transform attribute
3b	Model 1 with *cancer deaths* effects coded	-4793.420	8	9602.839	9638.068	No improvement in model fit versus Model 1
3c	Model 1 with *overdiagnosis* effects coded	-4791.498	8	9598.997	9634.225	No improvement in model fit versus Model 1
3d	Model 1 with *false positives* effects coded	-4789.235	8	9594.470	9629.699	Improved model fit versus Model 1 so transform attribute
3e	Model 1 with *false negatives* effects coded	-4789.215	8	9594.429	9629.658	Improved model fit versus Model 1 so transform attribute
**Best transformation for non-linear attributes**						
4a	Model 1 with *cancers diagnosed* transformed (quadratic)	-4796.742	6	9605.483	9631.904	Model 4c (cancers diagnosed transformed (piecewise – cut off at 48)) is the best fit
4b	Model 1 with *cancers diagnosed* transformed (log)	-4797.965	6	9607.930	9634.351	
4c	Model 1 with *cancers diagnosed* transformed (piecewise – cut off at 48)	-4785.390	7	9584.779	**9615.604**	
4d	Model 1 with *cancers diagnosed* transformed (piecewise – cut off at 49)	-4791.150	7	9596.299	9627.124	
4e	Model 1 with *false positives* transformed (quadratic)	-4796.292	6	9604.583	9631.005	No substantial improvement in model fit versus Model 1
4f	Model 1 with *false positives* transformed (log)	NA				
4g	Model 1 with *false positives* transformed (piecewise – cut off at 300)	-4793.24	7	9600.480	9631.305	
4h	Model 1 with *false positives* transformed (piecewise – cut off at 400)	-4797.326	7	9608.652	9639.477	
4i	Model 1 with *false negatives* transformed (quadratic)	-4834.9	6	9681.799	9708.221	No improvement in model fit versus Model 1
4j	Model 1 with *false negatives* transformed (log)	NA				
4k	Model 1 with *false negatives* transformed (piecewise – cut off at 10)	-4835.467	6	9682.933	9709.355	
4l	Model 1 with *false negatives* transformed (piecewise – cut off at 20)	-4835.914	6	9683.829	9710.250	

604 participants; 16,308 observations.

AIC: Akaike information criteria; asc: alternative specific constant; BIC: Bayesian information criteria (adjusted for sample size); DF: Degrees of freedom; LL: Log likelihood.

Table S5. Latent class analysis model selection for Samples A and B.

A. Selecting the best latent class analysis model for Sample A.

	Model description	N	LL	DF	AIC	BIC (n=N)	Class shares	Notes
**Number of classes**								
1a	Two classes	414	-2600.874	13	5227.747	5280.084	0.791, 0.209	Select 3 classes to balance gain in AIC/BIC and class sizes
1b	Three classes	414	-2519.143	20	5078.286	5158.803	0.508, 0.307, 0.185	
1c	Four classes	414	-2405.323	27	4864.645	4973.344	0.240, 0.267, 0.417, 0.077	
1d	Five classes	414	-2362.217	34	4792.435	4929.314	0.353, 0.204, 0.073, 0.232, 0.138	
**Optimum seed**								
2a	Seed 2	414	-2519.144	20	5078.288	5158.805	0.522, 0.292, 0.185	Select seed 3
2b	Seed 3	414	-2491.469	20	5022.937	5103.455	0.623, 0.079, 0.298	
2c	Seed 15, 40, 11	414	-2519.143	20	5078.286	5158.803	0.508, 0.185, 0.307	
**Class membership – single characteristics**								
3a	Age: older vs younger than 50 years	398	-2368.160	22	4780.319	4868.021	0.270, 0.079, 0.651	All characteristics improve model fit independently
3b	Sex: female vs male	403	-2424.228	22	4892.456	4980.432	0.433, 0.176, 0.391	
3c	Ethnicity: white vs ethnic minority or any other	401	-2416.539	22	4877.078	4964.945	0.179, 0.309, 0.512	
3d	Education: degree or above vs no degree	398	-2390.323	22	4824.645	4912.347	0.513, 0.143, 0.344	
3e	Self-reported SES decile: 1-3 (low) vs 4-10	389	-2337.682	22	4719.365	4806.564	0.498, 0.169, 0.333	
3f	Personal cancer history: yes vs no or NR	397	-2397.768	22	4839.535	4927.182	0.508, 0.176, 0.316	
3g	Previously screened: yes vs no or NR or not eligible	400	-2407.713	22	4859.425	4947.238	0.525, 0.151, 0.324	
3h	Likely to develop cancer: yes vs unlikely or unsure	389	-2309.821	22	4663.642	4750.841	0.266, 0.079, 0.655	
3i	Worried about cancer: yes vs no or NR	399	-2412.384	22	4868.768	4956.525	0.520, 0.163, 0.317	
**Class membership – multiple characteristics**								
4a	Age, SES, cancer likely	373	-2240.658	26	4533.317	4635.278	0.198, 0.334, 0.468	No attributes significant
4b	SES, cancer likely	377	-2221.431	24	4490.863	4585.237	0.267, 0.077, 0.656	Cancer likely significant
4c	Age, cancer likely	383	-2273.625	24	4595.250	4690.003	0.267, 0.077, 0.656	Cancer likely significant
4d	Sex, cancer likely	387	-2292.881	24	4633.763	4728.765	0.267, 0.077, 0.656	Cancer likely significant
4e	Ethnicity, cancer likely	385	-2316.428	24	4680.856	4775.734	0.183, 0.293, 0.525	No attributes significant
4f	Education, cancer likely	386	-2278.961	24	4605.922	4700.862	0.265, 0.077, 0.658	Cancer likely and education significant
								All characteristics improve model fit but only cancer likely and education predict class with statistical significance

414 participants; 11,178 observations.

AIC: Akaike information criteria; BIC: Bayesian information criteria (adjusted for sample size); DF: Degrees of freedom; LL: Log likelihood; N: number of participants; NR: not reported; SES: socioeconomic status.

B. Selecting the best latent class analysis model for Sample B.

	Model description	N	LL	DF	AIC	BIC (n=N)	Class shares	Notes
**Number of classes**								
1a	Two classes	604	-4007.571	15	8045.141	8111.195	0.251, 0.749	Select 3 classes to balance gain in AIC/BIC and class sizes
1b	Three classes	604	-3896.165	23	7838.329	7939.612	0.242, 0.585, 0.172	
1c	Four classes	604	-3766.344	31	7594.688	7731.199	0.097, 0.244, 0.176, 0.484	
1d	Five classes	604	-3732.989	39	7543.978	7715.718	0.186, 0.098, 0.081, 0.173, 0.462	
**Optimum seed**								
2a	Seed 2	604	-3896.165	23	7838.329	7939.612	0.242, 0.585, 0.172	Select seed 17
2b	Seed 17, 978	604	-3877.919	23	7801.838	7903.120	0.096, 0.660, 0.243	
**Class membership – single characteristics**								
3a	Age: older vs younger than 50 years	600	-3843.024	25	7736.047	7845.970	0.095, 0.657, 0.248	All characteristics improve model fit independently
3b	Sex: female vs male	602	-3855.023	25	7760.046	7870.052	0.097, 0.659, 0.244	
3c	Ethnicity: white vs ethnic minority or any other	599	-3838.394	25	7726.789	7836.670	0.096, 0.662, 0.242	
3d	Education: degree or above vs no degree	600	-3843.890	25	7737.779	7847.702	0.096, 0.658, 0.247	
3e	Self-reported SES decile: 1-3 (low) vs 4-10	593	-3809.933	25	7669.865	7779.495	0.096, 0.645, 0.259	
3f	Personal cancer history: yes vs no or NR	598	-3833.384	25	7716.768	7826.608	0.097, 0.657, 0.246	
3g	Previously screened: yes vs no or NR or not eligible	596	-3820.009	25	7690.018	7799.774	0.096, 0.659, 0.245	
3h	Likely to develop cancer: yes vs unlikely or unsure	568	-3622.310	25	7294.620	7403.173	0.093, 0.659, 0.248	
3i	Worried about cancer: yes vs no or NR	598	-3831.550	25	7713.100	7822.939	0.096, 0.659, 0.245	
**Class membership – multiple characteristics**								
4a	Age, SES, cancer likely	561	-3578.749	29	7215.497	7341.059	0.089, 0.656, 0.255	Age significant
4b	SES, cancer likely	562	-3587.039	27	7228.077	7345.028	0.092, 0.657, 0.251	No attributes significant
4c	Age, cancer likely	567	-3614.374	27	7282.748	7399.938	0.090, 0.658, 0.251	Age significant
4d	Sex, cancer likely	568	-3621.187	27	7296.374	7413.611	0.093, 0.659, 0.248	No attributes significant
4e	Ethnicity, cancer likely	566	-3608.040	27	7270.080	7387.223	0.093, 0.661, 0.246	No attributes significant
4f	Education, cancer likely	567	-3614.494	27	7282.987	7400.177	0.093, 0.657, 0.250	No attributes significant
4g	Age, sex	600	-3842.218	27	7738.437	7857.154	0.095, 0.657, 0.248	Age significant
4h	Age, ethnicity	597	-3825.113	27	7704.227	7822.809	0.094, 0.660, 0.246	Age significant
4i	Age, education	598	-3831.038	27	7716.077	7834.704	0.094, 0.656, 0.251	Age significant
4j	Age, SES	591	-3796.173	27	7646.346	7764.655	0.093, 0.655, 0.253	Age significant
								All characteristics improve model fit but only age predicts class with statistical significance

604 participants; 16,308 observations.

AIC: Akaike information criteria; BIC: Bayesian information criteria (adjusted for sample size); DF: Degrees of freedom; LL: Log likelihood; N: number of participants; NR: not reported; SES: socioeconomic status.

Table S6. Full results of latent class analysis and class membership for Samples A and B.

A. Sample A.

	Class 1A					Class 2A					Class 3A				
**Attribute**	**Coefficient**	**Lower CI**	**Upper CI**	**P value**		**Coefficient**	**Lower CI**	**Upper CI**	**P value**		**Coefficient**	**Lower CI**	**Upper CI**	**P value**	
**Class share**	65.8%					26.5%					7.7%				
**DCE attributes**															
No screening (ASC)	-4.211	-5.475	-2.947	<0.001	*	-1.133	-1.691	-0.574	<0.001	*	1.904	-1.008	4.816	0.200	
Cancers diagnosed	-0.079	-0.106	-0.051	<0.001	*	-0.068	-0.124	-0.011	0.020	*	-0.048	-0.227	0.130	0.596	
Cancer deaths	-0.295	-0.322	-0.268	<0.001	*	-0.420	-0.486	-0.353	<0.001	*	-0.375	-0.640	-0.110	0.006	*
Overdiagnoses	-0.008	-0.015	-0.001	0.021	*	-0.041	-0.056	-0.025	<0.001	*	-0.014	-0.061	0.032	0.552	
False positives	-0.002	-0.002	-0.001	<0.001	*	-0.004	-0.006	-0.003	<0.001	*	-0.006	-0.012	-0.001	0.021	*
False negatives	-0.020	-0.023	-0.016	<0.001	*	-0.041	-0.049	-0.034	<0.001	*	-0.051	-0.092	-0.011	0.013	*
**Class membership**															
Degree level education (vs no degree)	Ref					1.218	0.470	1.967	0.001	*	0.950	-0.122	2.022	0.083	
Consider likely to get cancer (vs unlikely)	Ref					-0.741	-1.334	-0.147	0.014	*	-0.990	-1.842	-0.139	0.023	*

**p<0.05; ASC: alternative specific constant; CI: 95% confidence interval.*

386 participants; 10,422 observations.

B. Sample B.

	Class 1B					Class 2B					Class 3B				
**Attribute**	**Coefficient**	**Lower CI**	**Upper CI**	**P value**		**Coefficient**	**Lower CI**	**Upper CI**	**P value**		**Coefficient**	**Lower CI**	**Upper CI**	**P value**	
**Class share**	65.7%					24.8%					9.5%				
**DCE attributes**															
No screening (ASC)	-2.776	-3.183	-2.369	<0.001	*	-0.067	-0.536	0.402	0.780		1.117	-0.683	2.916	0.224	
Cancers diagnosed – ≤48	-0.056	-0.091	-0.020	0.002	*	0.015	-0.052	0.083	0.656		0.074	-0.172	0.320	0.556	
Cancers diagnosed – >48	-0.216	-0.288	-0.144	<0.001	*	-0.262	-0.415	-0.110	0.001	*	-0.205	-0.771	0.362	0.479	
Cancer deaths	-0.278	-0.300	-0.257	<0.001	*	-0.374	-0.427	-0.321	<0.001	*	-0.309	-0.498	-0.121	0.001	*
Overdiagnoses	-0.006	-0.011	0.000	0.033	*	-0.017	-0.028	-0.006	0.003	*	-0.020	-0.054	0.014	0.256	
False positives	-0.001	-0.002	-0.001	<0.001	*	-0.004	-0.005	-0.003	<0.001	*	-0.010	-0.014	-0.006	<0.001	*
False negatives	-0.023	-0.026	-0.020	<0.001	*	-0.033	-0.039	-0.026	<0.001	*	-0.012	-0.031	0.007	0.206	
**Class membership**															
Age over 50 years (vs under 50)	0.612	0.185	1.039	0.005	*	Ref					0.813	0.147	1.478	0.017	

**p<0.05; ASC: alternative specific constant; CI: 95% confidence interval.*

600 participants; 16,200 observations.

Table S7. Participants' ease of completing the DCE and strategy for selecting their preferred options.

	Total sample		Sample A		Sample B		X^2 p-value for Sample A versus B*
	N	%	N	%	N	%	
**Total**	1018	100.0	414	40.7	604	59.3	
**Ease of completing the DCE**							<0.001
Difficult	711	69.8	330	79.7	381	63.1	
Easy	278	27.3	67	16.2	211	34.9	
Missing (not reported)	29	2.8	17	4.1	12	2.0	
**Strategy for selecting responses**							
**Strategy**							0.064
Considered all attributes	505	49.6	218	52.7	287	47.5	
Choices were based on 1/2 attributes	502	49.3	188	45.4	314	52.0	
Missing (not reported)	11	1.1	8	1.9	3	0.5	
**Attributes considered**							
Number of cancers prevented							0.275
Considered	686	67.4	287	69.3	399	66.1	
Not considered	332	32.6	127	30.7	205	33.9	
Number of deaths prevented							0.500
Considered	899	88.3	369	89.1	530	87.7	
Not considered	119	11.7	45	10.9	74	12.3	
People with overdetection and overtreatment							0.093
Considered	462	45.4	201	48.6	261	43.2	
Not considered	556	54.6	213	51.4	343	56.8	
People with false positives							0.662
Considered	535	52.6	221	53.4	314	52.0	
Not considered	503	49.4	213	221	290	48.0	
People with false negatives							0.850
Considered	687	67.5	278	67.1	409	67.7	
Not considered	331	32.5	136	32.9	195	32.3	

**Not including missing.*

Figure S1. Participants' ranking of the attributes after completing the DCE.

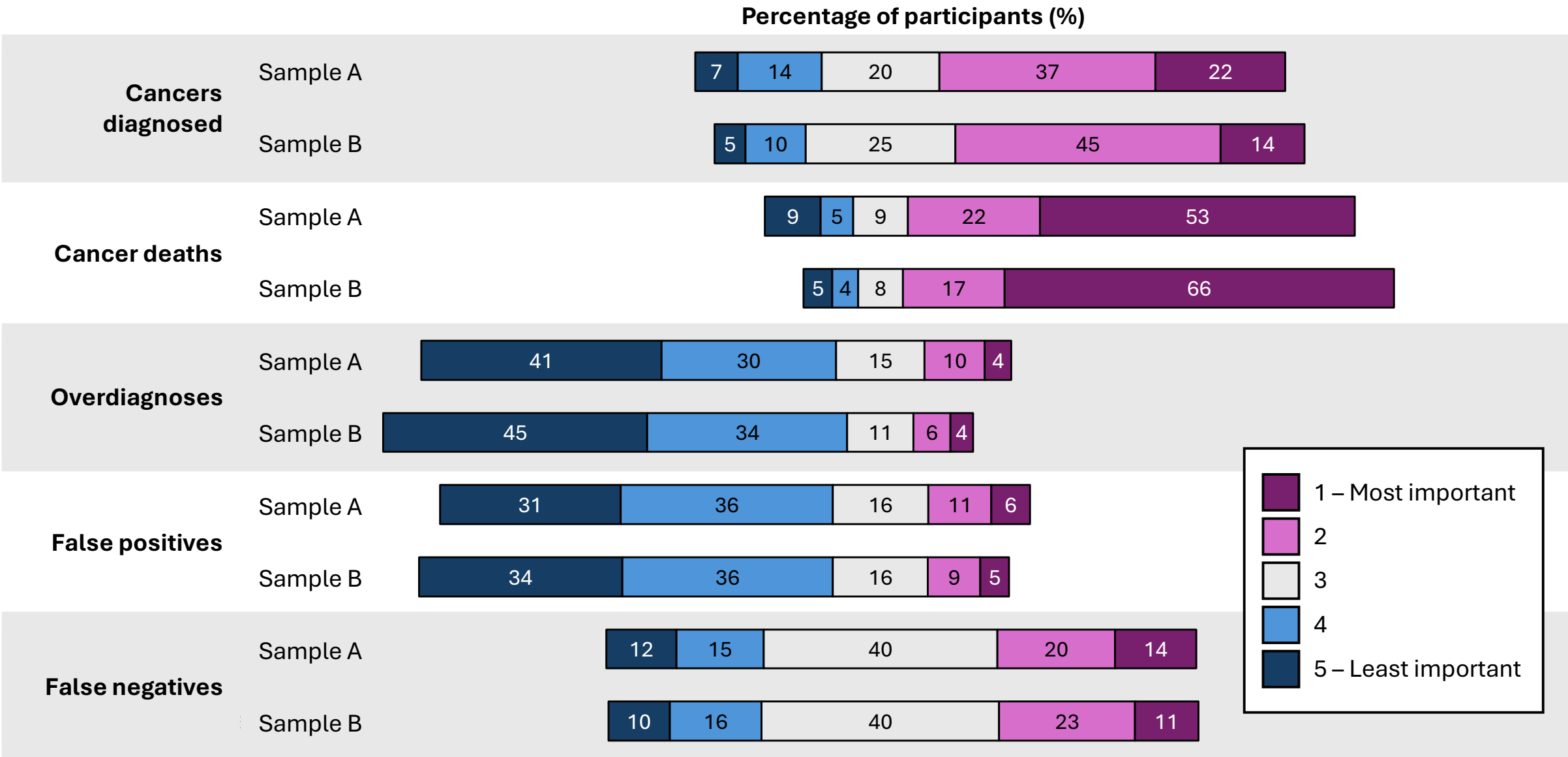

Rank	Cancers diagnosed		Cancer deaths		Overdiagnoses		False positives		False negatives	
	N	%	N	%	N	%	N	%	N	%
**Sample A (n=401)**										
**1 – most important**	88	21.9	214	53.4	18	4.5	26	6.5	55	13.7
**2**	147	36.7	90	22.4	41	10.2	43	10.7	80	20.0
**3**	80	20.0	37	9.2	60	15.0	65	16.2	159	39.7
**4**	57	14.2	22	5.5	119	29.7	144	35.9	59	14.7
**5 – least important**	29	7.2	38	9.5	163	40.6	123	30.7	48	12.0
**Sample B (n=596)**										
**1 – most important**	85	14.3	393	65.9	23	3.9	29	4.9	64	10.7
**2**	268	45.0	103	17.3	37	6.2	53	8.9	137	23.0
**3**	151	25.3	45	7.6	67	11.2	96	16.1	240	40.3
**4**	61	10.2	26	4.4	202	33.9	213	35.7	93	15.6
**5 – least important**	31	5.2	29	4.9	267	44.8	205	34.4	62	10.4
